# The Efficacy and Innocency of Solvents Candidly Examined; with Experiments and Cases

**Published:** 1783

**Authors:** 


VII. *The efficacy and innocency of Solvents candidly examined; with experiments and cafes. By* Robert Home, *furgeon to the Savoy.* 8vo. Murray, *London,* 1783, 78 pages, price 1s. 6d.

THE author of this little work having laboured under nephritic complaints fince the year 1768, has had recourfe to a variety of remedies, and among others to different folvents, the comparative effects of which he has endeavoured to afcertain by experiments.

The work is divided into three chapters.—In the firft the author relates his own cafe.—It feems that for feveral years after gravel had begun to form in his kidnies, he experienced confiderable relief from a liberal ufe of honey; but, at length, in 1780, he began to have fymptoms of of a ſtone in his bladder, which ſoon became ſo painful, that in May 1781, he was induced to try the effects of Dr. Jurin's lixivium. Of this medicine he took two tea ſpoonfuls twice a day in half a pint of veal broth. During the firſt days of this courſe the irritation of the bladder was much increaſed, but in a fortnight the ſymptoms were become very ſupportable. After perſevering in the uſe of this lixivium for the ſpace of two months, he changed it for Blackrie's, of which he took a tea ſpoonful twice a day till the middle of September, when his complaints were ſo much relieved, that he laid it aſide. At the end of ſix weeks, however, a return of the ſymptoms obliged him to have recourſe to it again, and it ſoon produced the ſame good effect as before. Relapſing again after a few weeks, he took two tea ſpoonfuls of Lane's lixivium twice a day, and this had the ſame effect as the others. He afterwards was deſirous of making a ſimilar trial with Adams's ſolvent; but the flavour of the different herbs made uſe of to diſguiſe it, and the opium which it appears to contain, occaſioned it to diſagree with him ſo much, that he determined to lay it aſide, and to return to the uſe of Blackrie's lixivium.

During

During the ufe of thefe different folvents, viz. Jurin's, Blackrie's and Lane's, Mr. Home made a variety of experiments, not only with feveral fmall calculi he himfelf had paffed, but with portions of larger calculi extracted from others by lithotomy. Thefe he immerfed in his urine, and from thefe experiments, which appear to have been carefully made, and candidly and accurately related, it appears that the urine of a perfon who is under a courfe of lixivium, poffeffes a folvent power.

At firft he was very careful to comply with the regimen recommended by Blackrie and Dr. Chittick, who, among other things, enjoin abftinence from falt. But it having been fuggefted to Mr. Home, that although a ftrong acid is extracted from fea falt by a chemical operation, it is fo concentrated in the falt, that the juices in the ftomach and inteftines cannot poffibly decompofe it, he ventured to eat not only falt, but likewife fat, greens, and every thing that was not directly acid, without finding the folvent power of the lixivium weakened by this deviation from the ufual regimen. During the ufe of this remedy the author tells us, that, excepting twice, when he thought it right to intermit termit taking the ſolvent for a few days on account of ſlight feveriſh ſymptoms, his appetite, digeſtion and general health have been perfectly good, although he is in an advanced age. He is now able to bear the motion of a carriage on the pavement, and to walk five or ſix miles without the ſmalleſt uneaſineſs. The only inconvenience of which he complains, is an inability to retain his urine (except when in bed) above two or three hours together. At the time of his writing this account of his caſe twenty months had elapſed ſince he began the uſe of the remedy, but he intended to perſevere in it for a conſiderable time longer, even though there ſhould be no apparent neceſſity for it.

In his ſecond chapter Mr. Home vindicates the innocency and efficacy of ſolvents from the cenſure thrown on them by Mr. Newman in his late publication on this ſubject *; and in the ſubſequent chapter we meet with ſeveral well atteſted inſtances of the beneficial effects of ſolvents, perfectly free from any bad conſequences.

The work concludes with ſome experiments on the comparative ſtrength of the lixivia al-

* See our 2d vol. p. 222.

ready mentioned; from which it appears, that the doses prescribed in the printed directions given with each of them, are properly enough adjusted to their strength, viz. of Blackrie's only one tea spoonful, of Jurin's and Lane's two, and of Adams's three tea spoonfuls twice a day.

